# Amino acid transport system - A substrate predicts the therapeutic effects of particle radiotherapy

**DOI:** 10.1371/journal.pone.0173096

**Published:** 2017-02-28

**Authors:** Tomoya Uehara, Mariko Watanabe, Hiroyuki Suzuki, Yoshiya Furusawa, Yasushi Arano

**Affiliations:** 1 Department of Molecular Imaging and Radiotherapy, Graduate School of Pharmaceutical Science, Chiba University, Chiba, Japan; 2 National Institutes for Quantum and Radiological Science and Technology, National Institute of Radiological Sciences, Chiba, Japan; Chiba Daigaku, JAPAN

## Abstract

L-[methyl-^11^C]Methionine (^11^C-Met) is useful for estimating the therapeutic efficacy of particle radiotherapy at early stages of the treatment. Given the short half-life of ^11^C, the development of longer-lived ^18^F- and ^123^I-labeled probes that afford diagnostic information similar to ^11^C-Met, are being sought. Tumor uptake of ^11^C-Met is involved in many cellular functions such as amino acid transport System-L, protein synthesis, and transmethylation. Among these processes, since the energy-dependent intracellular functions involved with ^11^C-Met are more reflective of the radiotherapeutic effects, we evaluated the activity of the amino acid transport System-A as an another energy-dependent cellular function in order to estimate radiotherapeutic effects. In this study, using a carbon-ion beam as the radiation source, the activity of System-A was evaluated by a specific System-A substrate, alpha-[1-^14^C]-methyl-aminoisobutyric acid (^14^C-MeAIB). Cellular growth and the accumulation of ^14^C-MeAIB or ^14^C-Met were evaluated over time *in vitro* in cultured human salivary gland (HSG) tumor cells (3-Gy) or *in vivo* in murine xenografts of HSG tumors (6- or 25-Gy) before and after irradiation with the carbon-ion beam. Post 3-Gy irradiation, *in vitro* accumulation of ^14^C-Met and ^14^C-MeAIB decreased over a 5-day period. In xenografts of HSG tumors in mice, tumor re-growth was observed *in vivo* on day-10 after a 6-Gy irradiation dose, but no re-growth was detected after the 25-Gy irradiation dose. Consistent with the growth results, the *in vivo* tumor accumulation of ^14^C-MeAIB did not decrease after the 6-Gy irradiation dose, whereas a significant decrease was observed after the 25-Gy irradiation dose. These results indicate that the activity of energy dependent System-A transporter may reflect the therapeutic efficacy of carbon-ion radiotherapy and suggests that longer half-life radionuclide-labeled probes for System-A may also provide widely available probes to evaluate the effects of particle radiotherapy on tumors at early stage of the treatment.

## Introduction

The use of particle beams such as carbon-ions and protons have several advantages over conventional X- or γ-ray beam radiotherapies, the most prominent of which is a lower radiation dose delivered to normal tissues surrounding the tumor [1]. Moreover, other potential advantages over the use of X- or γ-ray radiotherapies include a higher relative biological effectiveness (RBE) and a lower oxygen enhancement ratio [1, 2]. More than 50 centers currently use particle beams for radiotherapy with other centers under construction [1, 3–5]. Although particle beams have a relatively higher RBE and display less difference in sensitivity between the cell lines, compared with X- or γ-ray beams, the radiotherapeutic response of tumors post beam-irradiation differed from the cell-lines or the tumor site [1, 6]. In clinical studies, tumor recurrences post-irradiation with particle beams have been also observed [1, 7]. Thus, an effective non-invasive means to evaluate the therapeutic efficacy of particle beam radiotherapies, as well as X- or γ-ray radiotherapy, early in the treatment protocol would be valuable.

Functional imaging with positron emission tomography (PET) using radiopharmaceuticals such as 2-[^18^F]fluoro-2-deoxy-D-glucose (^18^F-FDG) and L-[methyl-^11^C]methionine (^11^C-Met) reflects the biochemical and physiological characteristics of tumors [8, 9]. Thus, this imaging technique provides an earlier assessment of response to chemotherapy for cancer more than a structural or morphological diagnosis by X-ray computed tomography (CT) and magnetic resonance imaging (MRI) [10–12]. Since activated inflammatory immune cells are present and recruited at the irradiation sites post-irradiation and these cells also incorporated ^18^F-FDG, which frequently occurs, this complicates diagnostic accuracy [13, 14]. ^11^C-Met is, therefore, frequently used to estimate the therapeutic efficacy of particle beam radiotherapy [7, 12, 15]. However, due to the short half-life (20 min) of the ^11^C-isotope, adjacency to GMP-capable cyclotron facilities is required to produce and use ^11^C-Met for clinical practice [16]. Thus, the development of a longer half-life ^18^F-, ^76^Br, ^123^I-, or other radionuclide-labeled probes that provide a post-irradiation response similar to that observed with ^11^C-Met are being investigated and pursued.

The uptake of ^11^C-Met by tumor cells is multifaceted, as methionine is involved in many cellular functions such as a substrate for amino acid transporters (mainly *via* the L-type amino acid transporter 1 (LAT1) [17, 18]), protein synthesis, and transmethylation [8, 19, 20]. Among these roles, it is the energy-dependent intracellular functions, such as protein synthesis and transmethylation, that are more reflective of the therapeutic effects of carbon-ion radiotherapy rather than the energy-independent amino acid transport System-L function that is involved in the transport of ^11^C-Met [21]. Since a part of the intracellular metabolism of methionine is structurally methionine-specific (e.g. transmethylation), it is deemed difficult to develop longer lived radioisotope such as ^18^F, ^76^Br, and/or ^123^I-labeled probes that reflect this particular energy-dependent aspect of the intracellular metabolism of ^11^C-Met.

Amino acids are transported into cells via the amino acid transport systems, which have been classified into 2 groups [20]. Transporters in the first group function in a sodium-independent manner, and contain the amino acid transport System-L. This energy-independent System-L transporter is involved in the transport of methionine into the cell. Transporters in the second group require a sodium chemical gradient and the membrane electric potential for their functional activity, and contain the amino acid transport System-A [20, 22, 23]. The driving force that energizes this type of transporter is maintained by Na^+^/K^+^ ATPase. Thus, the System-A transporter is energy-dependent transporter while System-L is energy-independent transporter [24, 25]. And several ^18^F- and ^123^I-labeled labeled probes that are transported into cells via this amino acid transport System-A have been developed as a tumor-imaging agent [26–30].

Since the energy-dependent intracellular metabolic processes that ^11^C-Met is involved in are reflective of radiotherapeutic response [21], we hypothesized whether the energy-dependent activity of the System-A transporter could be used to predict the radiotherapeutic efficacy of a carbon-ion beam at an early stage of the treatment process. The artificial amino acid, alpha-(methylamino)isobutyric acid (MeAIB), represents a standard substrate for all three system A subtypes; SNAT1, SNAT2, and SNAT4 [23, 31], and is not metabolized within cells [27]. Thus, the accumulation of MeAIB in tumor cells may reflect the energy-dependent activity of System-A. In this context, we evaluated the activity of the energy-dependent amino acid transport System-A with ^14^C-MeAIB in tumor cells post irradiation with a carbon-ion beam and assessed the potential of substrates for this energy-dependent System-A transporter to reflect the radiotherapeutic efficacy of the radiotherapy. As the short half-life of ^11^C-labeled Met would preclude its suitability for these studies, ^14^C-labeled analogue was used instead, in conjunction with the Heavy Ion Medical Accelerator in Chiba (HIMAC) synchrotron at National Institute of Radiological Sciences (NIRS) in Japan.

## Materials and methods

### General

Alpha-[1-^14^C]-methylaminoisobutyric acid (^14^C-MeAIB, specific activity: 1.87 GBq/mmol) was purchased from Perkin Elmer Japan (Yokohama, Japan) and L-[methyl-^14^C]methionine (^14^C-Met, specific activity: 2.22 GBq/mmol) was purchased from GE Healthcare (Tokyo, Japan). Radioactivity counts were determined using a liquid scintillation counter (LSC-5100, Aloka, Tokyo). ATP was measured by a luminometer (TD-20/20, Turner Designs, CA). Four-week-old male Balb/cAJcl-nu nude mice (Nihon Clea, Tokyo, Japan) were used in the *in vivo* studies. Other materials reagents were of reagent grade and used as received.

### Irradiation

Carbon-ion beams were accelerated up to 290 MeV/u by HIMAC synchrotron at NIRS in Japan. Modulator films were used to achieve an LET value of 60 keV/μm within a 6-cm-wide spread-out-Bragg-peak (SOBP). All samples were irradiated at room temperature.

### Cell culture

The human salivary gland (HSG) cell line [32] was generous gifts from NIRS. The HSG cell line has previously been used and described in preclinical radiobiologic experiments with the carbon-ion beams at NIRS [33, 34]. Moreover, the ridge filter in the instrument was designed so that the HSG cells were uniformly irradiated at the SOBP in the carbon-ion beam at NIRS [35]. Based on these prior experiments, the HSG cell line was used for these studies. The HSG cell was grown as a monolayer in Eagle’s Minimum Essential Medium (EMEM, Sigma-Aldrich Japan, Tokyo), supplemented with 10% fetal bovine serum (SAFC Biosciences, Kansas) and 1% penicillin/streptomycin (10,000 units/mL, 10 mg/mL) at 37°C in a 5% CO_2_ atmosphere. Prior to the cell uptake experiments, HSG cells were washed twice with PBS and isolated as a single-cell suspension by trypsinizing with a solution of 0.25% trypsin in PBS. After the cells were resuspended in EMEM medium, cell numbers were counted using a Coulter counter (Beckman Coulter, Tokyo) and the cell concentration was adjusted to the desired concentration.

### Cell uptake experiments

Cell uptake experiments were performed according to the procedure described by Samnick et al. [36] with slight modifications. The cell suspension was transferred to a 15-mL tube and then centrifuged for 5 min at 1,000 x *g*. The resulting supernatant was removed, and the pellet was washed with 2 mL of HEPES buffer (pH 7.4, 12 mM HEPES, 137 mM NaCl, 2.7 mM KCl, 1 mM MgCl_2_, and 5.6 mM D-glucose). To conduct the Na^+^-free uptake studies, NaCl was replaced with choline-Cl (Na^+^-free HEPES buffer). The cell suspension (500 μL) was transferred to 2-mL Eppendorf tubes at concentrations of 2 x 10^6^ cells/mL for experiments and preincubated at 37°C for 15 min. A 20-μL aliquot of ^14^C-compound (^14^C-MeAIB, 5.18 x 10^2^ Bq, or ^14^C-Met, 6.29 x 10^2^ Bq, in HEPES buffer) with or without amino acid transport System-A or L inhibitors (5 mM MeAIB or 5 mM 2-amino-2-norbornane carboxylic acid (BCH), respectively) was added to the cells, which were then incubated at 37°C or 4°C for 5 min. The tracer uptake was stopped by the addition of 1 mL of ice-cold PBS and an additional 2 min in an ice bath, followed by centrifuging for 2 min at 1,000 x *g*. The resulting supernatant was removed, and the cell-pellet was washed three times with ice-cold PBS. Radioactivity was measured by first dissolution of the pellet in a tissue solubilizer (0.5 mL, SOLUENE-350, Perkin Elmer Japan), followed by the addition of a scintillator (5 mL, HYONIC-FLUOR, Perkin Elmer Japan) and counted on an automated liquid scintillation counter.

### Cell uptake experiments after irradiation

Harvested and EMEM washed HSG tumor cells were counted using a Coulter counter and the cell concentration was adjusted to 1 x 10^6^ cells or 2 x 10^6^ cells that were plated in T-25 flasks. These flasks were incubated at 37°C in 5% CO_2_ for 1 day. The cell bearing flasks were then irradiated with a carbon-ion beam to attain a dose of 3-Gy. Post-irradiation, the media in each flask was changed to fresh media, and flasks were incubated for the appropriate time for subsequent amino acid uptake studies conducted on the assigned day. On the study day, the cells were washed, harvested and counted using a Coulter counter to provide the surviving tumor cell number. The cell concentration then was adjusted to 2 x 10^6^/mL and the cell uptake of ^14^C-MeAIB or ^14^C-Met by HSG cells was investigated from 1–5 days post-irradiation using the procedure described above.

### ATP content in cells after irradiation

Irradiated cells were prepared as described above. The cell suspension was transferred to a 15-mL tube and centrifuged at 1,000 x *g* for 5 min. The resulting supernatant was removed, and the pellet was washed twice with PBS and resuspended in PBS to a concentration of 3 x 10^5^ cells/mL. A cellular ATP measurement kit for cells (Cosmo bio Ltd., Tokyo) was used to measure the ATP content of the cells, following the manufacturer’s protocol. The content of ATP was measured using the chemiluminescence produced by the luciferase/luciferin reaction [37].

### *In vivo* experiments

HSG cells (1 x 10^6^ cells) were transplanted subcutaneously to the left hind-limbs of nude mice 15 days prior to irradiation. Before irradiation, the tumor volumes were measured and conformed to 1.25 ± 0.18 cm^3^. After the tumors reached an appropriate size, under pentobarbital anesthesia (20 mg/kg) each mouse was immobilized on a Lucite plate and its left hind leg was placed in the irradiation field. In separate groups of mice, the implanted HSG tumors received a radiation dose of 6-Gy or 25-Gy from the carbon-ion beam. To investigate the uptake of ^14^C-MeAIB or ^14^C-Met at the irradiation site, the left hind leg (muscle) was also irradiated by the carbon-ion beam (25-Gy) as described above. The rate of tumor growth was assessed by daily measurements of tumor sizes from which estimates for tumor volume (*v*) were made using the formula *v* = *a* x *b* x *c* (where *a*, *b*, and *c* are tumor diameters). ^14^C-MeAIB (11.1 kBq) or ^14^C-Met (11.1 kBq) dissolved in saline was administrated intravenously before mice were sacrificed at 30 min. Tissues of interest were removed and weighed. The tissues were then solubilized in soluene-350 and Hionic-Fluor and radioactivity was measured using a liquid scintillation counter. Values were expressed as the mean (SD) for a group of 3–5 animals.

### Statistical analysis

Results are expressed as the mean ± SD. Results were analyzed using the Tukey’s multiple-comparison test (Graph Pad Prism, CA) to compare the significance of differences between two groups. Differences were considered significant when *p* values were less than 0.05.

### Ethical approval

All animal studies were conducted in accordance with institutional ethical guidelines and were approved by the Chiba University Animal Care Committee. We regularly monitored each animal’s health at every 2–3 days. If the tumor volumes in any animals were over 2.5 cm^3^, the individual animal was euthanized by decapitation. There are no mice that became ill or died at any time prior to the experimental endpoint.

## Results

### Transport mechanisms of ^14^C-MeAIB and ^14^C-Met in HSG cells

**Fig 1:**
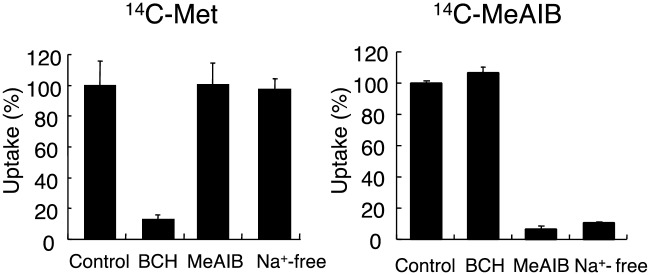
Experimental definition of ^14^C-Met and ^14^C-MeAIB transport systems in HSG cells. The uptake of ^14^C-Met (left) and ^14^C-MeAIB (right) was measured in the presence or absence of 5 mM BCH, 5 mM MeAIB, or Na^+^. Values are the mean ± SD.

The tumor cell uptake of ^14^C-Met was not reduced in Na^+^-free buffer or in the presence of MeAIB, a substrate of amino acid transport System-A. However, a significant reduction (13% of the control) was observed in the presence of BCH, a standard inhibitor of amino acid transport System-L (Fig 1). On the other hand, the uptake of ^14^C-MeAIB in Na^+^-free buffer was reduced to 11% of the control and was inhibited by MeAIB to 6% of the control, but was not affected by BCH (Fig 1).

### Cell numbers, tumor uptakes of ^14^C-MeAIB and ^14^C-Met, and ATP contents in cells after irradiation

**Fig 2:**
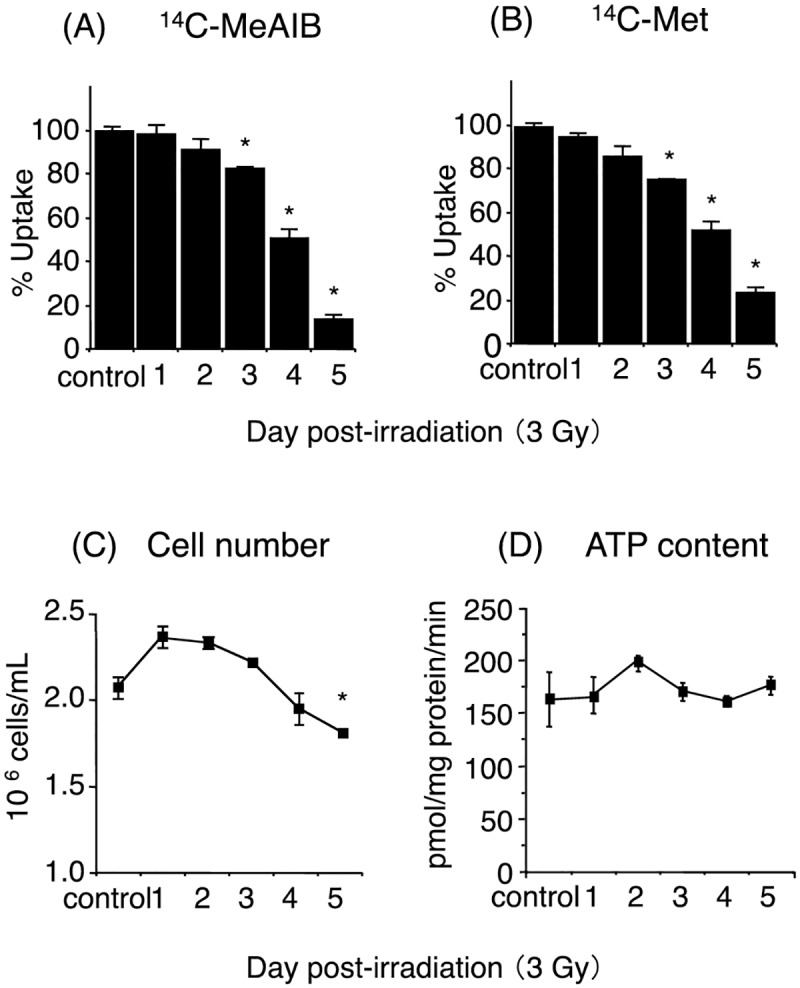
Time course of changes after irradiation by the carbon-ion beam (3-Gy) on the *in vitro* uptake of ^14^C-MeAIB (A) and ^14^C-Met (B) by HSG cells, and effects on the number (C) and ATP content (D) of HSG cells. Values are the mean ± SD. Significant differences were determined by Tukey’s multiple-comparison test *p*<0.05 vs. non-irradiation.

The time course of changes in the cell numbers after irradiation by the carbon-ion beam (3-Gy) is shown in Fig 2C. The cell growth was measured as cell numbers, and a slight increase was observed 1 day post-irradiation, which subsequently decreased with time, with a significant decrease in the cell population observed 5-days post-irradiation (*p*<0.05). In irradiated cells, the accumulation of ^14^C-Met was reduced to 96%, 75%, and 24% of the controls at 1-, 3-, and 5-days post-irradiation, respectively, and was significantly reduced from day-3 onward (*p*<0.05) (Fig 2B). Similarly, accumulation of ^14^C-MeAIB by the irradiated cells was reduced to 98%, 83%, and 13% of the controls at 1-, 3-, and 5-days post-irradiation, respectively, and was also significantly reduced from day-3 onward (*p*<0.05) (Fig 2A). The ATP content in all of these HSG cells was also measured post-irradiation (Fig 2D), where no significant changes were observed over the 5-day post-irradiation period.

**Fig 3:**
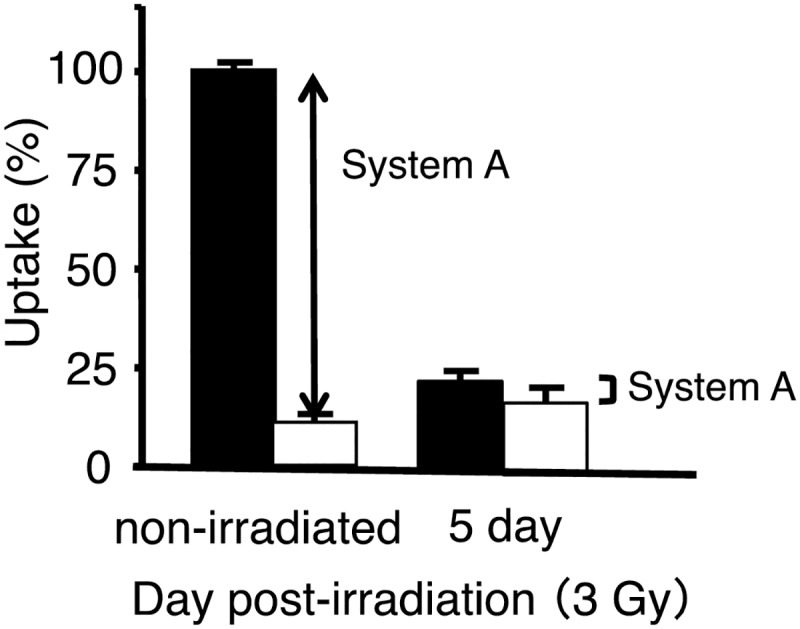
The uptake mechanisms of ^14^C-MeAIB pre- and post-irradiation with the carbon-ion beam. The total uptake of ^14^C-MeAIB was divided into its transport system contributions before and after irradiation to HSG cells by the carbon-ion beam as follows: System-A, the part of uptake that is inhibited by 5 mM MeAIB. The uptake of ^14^C-MeAIB was measured in the presence (white bar) or absence (black bar) of 5 mM MeAIB. Values are the mean ± SD.

Fig 3 illustrated the uptake of ^14^C-MeAIB by HSG tumor cells pre- and post-irradiation with the carbon-ion beam (3-Gy). The results obtained, show that the uptake of ^14^C-MeAIB by pre-irradiated cells in the presence of 5 mM MeAIB was significantly lower than that of the control. However, when similar uptake measurements of ^14^C-MeAIB were performed 5 days post-irradiation with the carbon-ion beam at a dose of 3-Gy, the cellular uptake of the System-A component was significantly reduced.

#### *In vivo* experiments

**Fig 4:**
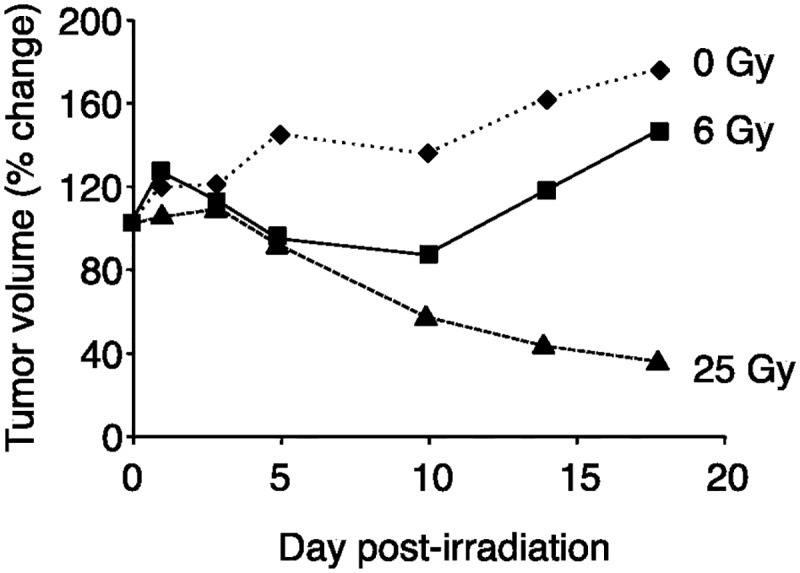
Time course of changes in tumor volumes after irradiation by the carbon-ion beam (0 (diamond), 6 (square), or 25 Gy (triangle)). With the 6-Gy dose, tumor volume reductions were observed and were followed by a re-growth. The irradiation of tumors with the 25-Gy dose resulted in a significant reduction in tumor size, with no tumor re-growth.

*In vivo* tumor growth (tumor volume) curves after irradiation with the carbon-ion beam with a 6- or 25-Gy irradiation dose are shown in Fig 4 and S1 Fig. With the 6-Gy dose, tumor volume reductions were observed for up to 10 days post-irradiation, and were followed by a re-growth and significant volume increases (150% of the control by day 18). On the other hand, the irradiation of tumors with the 25-Gy dose resulted in an initial tumor volume increase 3 days post-irradiation, that was followed by a significant reduction in tumor size, with no tumor re-growth being detected up to 18 days post-irradiation.

**Fig 5:**
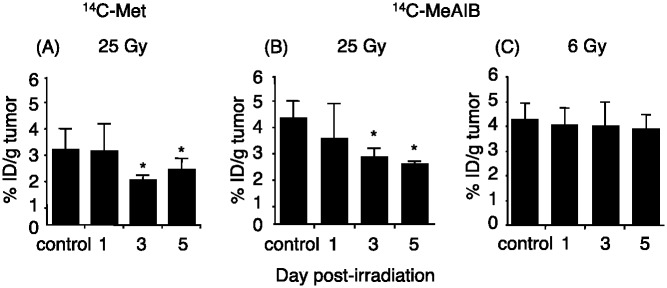
Time course of changes in the uptake of ^14^C-Met and ^14^C-MeAIB in HSG tumors after irradiation by the carbon-ion beam (6- or 25-Gy). Values are the mean ± SD. Significant differences were determined by Tukey’s multiple-comparison test *p*<0.05 vs. non-irradiation.

**Fig 6:**
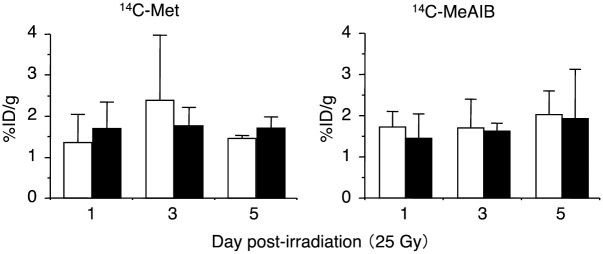
*In vivo* uptake of ^14^C-Met and ^14^C-MeAIB at irradiation sites (solid bar) post-irradiation with the carbon-ion beam (25-Gy) and at a non-irradiated site (white bar). Both ^14^C-Met and ^14^C-MeAIB displayed similar tissue accumulation values at the irradiated and non-irradiated muscle tissues. Values are the mean ± SD.

Measurements of the *in vivo* tumor uptake of ^14^C-Met by HSG tumor xenografts pre- and post-irradiation with the 25-Gy dose revealed a significant decrease in the accumulation of ^14^C-Met 3-days post-irradiation (Fig 5A; 60% from pre-irradiation). Similarly, the *in vivo* tumor accumulation of ^14^C-MeAIB was also significantly decreased 3-days post-irradiation with a 25-Gy dose (Fig 5B; 63% from pre-irradiation). When HSG tumor xenografts were irradiated with a lower dose (6-Gy), the tumor accumulation of ^14^C-MeAIB was not significantly reduced 5-days post-irradiation, and the tumor accumulation values of ^14^C-MeAIB at that time were 90% those of the pre-irradiation values (*p*>0.05) (Fig 5C). Fig 6 illustrates that both ^14^C-labeled compounds displayed similar tissue accumulation values at the irradiated and non-irradiated muscle tissues.

## Discussion

^11^C-Met is a useful radiopharmaceutical to estimate the therapeutic efficiencies of radiotherapy such as conventional and particle radiotherapies [12, 15, 38, 39]. Indeed, ^11^C-Met has been used to estimate the therapeutic efficacy of carbon-ion radiotherapy at NIRS. Among the cellular functions involved in ^11^C-Met such as transport (System-L), protein synthesis, and transmethylation [8, 17–20], energy-dependent intracellular functions are more reflective of the therapeutic effects of carbon-ion radiotherapy [21] and conventional radiotherapy [40, 41]. Although a number of radiolabeled amino acid analogues using ^18^F and ^123^I have been developed and reported to display tumor uptake via this energy-independent transport System-L [20, 29, 42, 43], these reagents have not been shown to provide information on subsequent intracellular energy-dependent processes similar to those observed with ^11^C-Met, e.g. protein synthesis, and/or transmethylation, nor have they been evaluated in the radiotherapy studies at early stage of the treatment. It is with these considerations, herein, we evaluated whether the activity of the amino acid transport System-A (an energy-dependent transport system) may have a potential utility in estimating the radiotherapeutic effect at early stage of the treatment, analogues to those observed with ^11^C-Met.

The results of the *in vitro* cell uptake studies clearly showed that the cellular uptake of ^14^C-MeAIB was significantly reduced under Na^+^-free conditions and was inhibited by MeAIB (>90%), but not by BCH (Fig 1), indicating that ^14^C-MeAIB was transported into HSG cells via an energy-dependent amino acid transport System-A. The uptake of ^14^C-Met by HSG tumor cells was inhibited by BCH (>80%) (Fig 1), but was not significantly affected by Na^+^-free conditions, indicating that ^14^C-Met was mainly transported into HSG cells via amino acid transport System-L. Taken together, these results indicate that the two amino acids, ^14^C-MeAIB and ^14^C-Met are transported into HSG cells by different transport systems and reflect energy-dependent and energy-independent transports into HSG tumor cells, respectively.

Fractionated irradiation is typically used during radiation treatment to improve the therapeutic ratio between target and normal tissue. Given the nature of the carbon-ion radiotherapy, the normal healthy cells surrounding the target area generally will receive a low dose of low-LET radiation and can escape from radiation-damage, while the LET of the therapeutic carbon-ion beams in target area remains effective [44], thus the advantage gained by fractionation of a carbon-ion beam irradiation is significantly enhanced [36]. Considering this and because this study focused on the tumor responses post-irradiation of carbon-ion beam, single irradiations were used for both *in vitro* and *in vivo* studies.

The *in vitro* cell survival after 3-Gy of carbon-ion irradiation was reported earlier to be 5% and less than 0.01% at survival was observed at 6-Gy irradiation determined by a colony formation assay [6]. In addition to this, the observed pattern in the reduction in cell-numbers observed *in vitro* 5 days post 3-Gy irradiation (Fig 2C), was similar to the reduction in tumor volume *in vivo* 5 days post 6-Gy irradiation (Fig 4), hence a 3-Gy irradiation dose was chosen for the *in vitro* cellular responses studies. An examination of the effects of the carbon-ion beam (6-Gy or 25-Gy) on the tumor cell survival in an *in vivo* systems showed that no significant changes occurred in tumor volumes for up to 5 days post-irradiation. However, the sizes of tumors irradiated with the lower dose (6-Gy) displayed an increase by 10-day, while those of tumors irradiated with a higher dose (25-Gy) continued to decrease in volume. Under these conditions, ^14^C-MeAIB uptake by tumor tissues post 6-Gy irradiation did not change up to 5 days, while the uptake and accumulation of ^14^C-MeAIB by tumor tissues irradiated with 25-Gy displayed significant decrease 3 days post-irradiation (Fig 5). The short half-life tracer ^11^C-Met has been used to estimate the therapeutic efficiencies of particle radiotherapy in clinical practice [12, 15] and a decrease in the uptake of ^11^C-Met has been shown to represent a positive treatment effect [45]. Thus, these results suggested that early changes in the uptake of ^14^C-MeAIB may also have a potential to display an early effect of the carbon-ion beam dose and tumor responses to this radiotherapy.

Radiation-induced cell death has been functionally classified into “interphase death” and “reproductive death” [46]. Interphase cell death is the death of irradiated cells prior to mitosis, while reproductive death is observed after several cell division cycles. Consistent with this, the morphological changes observed *in vitro* and *in vivo* post-irradiation with the carbon-ion beam also displayed a temporarily increase in cell numbers and tumor volume, and these were followed by decreases in both these parameters as dead tumor cells were eliminated by macrophages (Figs 2 and 4). When considering the cellular transport and metabolic functional changes occurring post-irradiation with the carbon-ion beam, we observed a reduction in the accumulation of ^14^C-MeAIB and ^14^C-Met in tumors prior to reductions in tumor volumes (Figs 2 and 5). Accumulation of ^14^C-MeAIB and ^14^C-Met at the normal tissue site (muscle) that was irradiated was not observed (Fig 6). Both ^14^C-MeAIB and ^14^C-Met displayed decreased accumulation in the tumor post irradiation (Figs 2 and 5), despite the two amino acids being transported into HSG cells via different transport systems (Fig 1). We have previously described that the decreased accumulation of ^14^C-Met was a reflection of a reduction in the energy-dependent intracellular metabolism of ^14^C-Met rather than a disruption in energy-independent System-L transporter functions [21]. Our findings here are consistent with those studies and additionally suggest that while the decrease observed in the accumulation of ^14^C-Met is due to energy-dependent intracellular metabolic processes for which ^14^C-Met is a substrate [21], reductions in the uptake of ^14^C-MeAIB were attributable to the disruptions in energy-dependent amino acid transport System-A (Fig 3). This is further supported by the *in vitro* studies, where the effects of carbon-ion beam irradiation on the content of ATP showed that the total cellular ATP levels did not change post-irradiation (Fig 2D); however, the uptake of ^14^C-MeAIB and ^14^C-Met by these exposed tumor cells was reduced over time post-irradiation (Fig 2A and 2B). These results indicate that carbon-ion beam irradiation resulted in cellular damage that significantly affected a number of metabolic pathways as well as transporter proteins, and/or the energy in the cell may be directed toward other functions such as DNA repair [47]. Taken together, these results suggest that the irradiation of HSG tumor cells affects energy-dependent processes prior to morphological changes, and in the case of ^14^C-MeAIB, the energy-dependent System-A transporter was affected, while in the case of ^14^C-Met, intracellular energy-dependent metabolic processes were disrupted, both of which result in displaying a decreased accumulation of the respective amino acid tracer at an early point in the treatment.

Most of the clinically established amino acid tracers such as ^18^F-FET, ^18^F-FMT and ^18^F-FBPA are taken up by tumors predominantly via the energy-independent amino acid transport System-L [9, 20, 43], similarly several ^18^F- and ^123/131^I-labeled amino acid tracers targeting the energy-dependent amino acid transport System-A such as ^18^F-FAMP, ^18^F-MeFAMP and ^123/131^I-VAIB and have also been developed [29, 30], however, very few of these have been evaluated in human studies for monitoring the dose-response to radiotherapy. Our investigation and data suggested that the energy-dependent amino acid transport Systems-A may indeed be use as a target to evaluate radiolabeled amino acid substrates and to monitor the therapeutic efficacy of the particle beam radiotherapy at early stage of the treatment.

## Conclusions

The results of the present study indicate that the activity of amino acid transport System-A reflects the therapeutic efficacy of particle radiotherapy at an early stage of the treatment, similar to that observed for the energy-dependent intracellular metabolism of ^14^C-Met. Given these findings, several longer half-life ^18^F- and ^123^I-labeled probes developed for the energy-dependent amino acid transport System-A may also have a potential utility and be more practical (longer half-life, and more widely available) as imaging probes to evaluate the effect of particle beam radiotherapy for tumors at an early stage of the treatment regimen.

## Supporting information

S1 Fig Time course of changes in tumor volumes (cm^3^) after irradiation by the carbon-ion beam (0 (diamond), 6 (square), or 25 Gy (triangle)). With the 6-Gy dose, tumor volume reductions were observed and were followed by a re-growth. The irradiation of tumors with the 25-Gy dose resulted in a significant reduction in tumor size, with no tumor re-growth.
(DOCX)
