# Supplementary material for: Amino acid transport system - A substrate predicts the therapeutic effects of particle radiotherapy
Source: PLoS One. 2017 Feb 28;12(2):e0173096. doi: 10.1371/journal.pone.0173096 (PMC5330493; doi:10.1371/journal.pone.0173096)
Supplement: S1 Fig — With the 6-Gy dose, tumor volume reductions were observed and were followed by a re-growth. The irradiation of tumors with the 25-Gy dose resulted in a significant reduction in tumor size, with no tumor re-growth. (DOCX) [file pone.0173096.s001.docx]

Supporting Information

**S1 Fig. Time course of changes in tumor volumes (cm^3^) after irradiation by the carbon-ion beam (0 (diamond), 6 (square), or 25 Gy (triangle)).** With the 6-Gy dose, tumor volume reductions were observed and were followed by a re-growth. The irradiation of tumors with the 25-Gy dose resulted in a significant reduction in tumor size, with no tumor re-growth.
